# ALT-tomato: a process-based model for Alternaria disease complex addressing mycotoxin risk

**DOI:** 10.3389/fpls.2026.1794763

**Published:** 2026-03-26

**Authors:** Irene Salotti, Marco Camardo Leggieri, Paola Battilani

**Affiliations:** 1 Department of Sustainable Crop Production (DI.PRO.VES.), Universitá Cattolica del Sacro Cuore, Piacenza, Italy; 2 Research Center on Plant Health Modelling (PHeM), Universitá Cattolica del Sacro Cuore, Piacenza, Italy

**Keywords:** botanical epidemiology, brown spot, disease modeling, early blight, model validation, system analysis

## Abstract

The Alternaria disease complex has emerged as a major concern in tomato cropping systems, posing significant threats to both tomato production and human health due to the co-occurrence of multiple species on the same plants and the production of mycotoxins. Predictive tools are instrumental in advancing food security and mitigating risks to food safety. Therefore, this study aimed to synthesize current knowledge on *Alternaria* spp. affecting tomato and develop a mechanistic, weather-driven model capable of predicting both epidemics and mycotoxin contamination. A systematic literature review was conducted to compile quantitative information on the ecology, biology, epidemiology, and mycotoxin production of *Alternaria* species associated with the disease complex. These data were used to construct a logical and mathematical framework describing interactions among major *Alternaria* species, tomato, and environmental drivers. The resulting model comprises interconnected compartments representing: (i) conidia production from overwintering and in-season sources, (ii) conidia dispersal, (iii) infection by conidia, (iv) symptom development, and (v) mycotoxin (alternariol, alternariol monomethyl ether, and tenuazonic acid) production and accumulation within tomato fruit tissues. Model parameterization was performed for three major species affecting tomato (*A. alternata*, *A. solani*, and *A. tenuissima*). Model validation using eight epidemics recorded in Italy, India, and Canada demonstrated strong agreement between predicted and observed outcomes, with a high concordance (CCC = 0.98) and a low average prediction error (RMSE = 0.069). Results suggest that the proposed framework may capture the complexity of the *Alternaria*-tomato pathosystem and could serve as the basis for a tool to support more informed and sustainable disease management strategies; however, its applicability to mycotoxin risk management remains to be validated.

## 1 Introduction

The call for a sustainable and safe agricultural production fosters the uptake of novel decision-making tools for plant disease management, including predictive models (González-Domínguez et al., 2023; Cunniffe et al., 2015; Gilligan, 2008). In fact, the prediction of disease occurrence with a long enough lead time may help farmers to implement control measures timely and efficiently (Rossi et al., 2023). Moreover, disease prediction has a potential in monitoring and managing food contaminants that threaten consumer health, such as mycotoxins (Casu et al., 2024; Battilani, 2016). Although predictive models have been built since the 1950s, trends in their development are influenced by numerous factors that comprise the emergence of diseases, new epidemiological knowledge, as well as public concerns on pathogen-related hazards (Mulligan and Wainwright, 2013; De Wolf and Isard, 2007).

Recently, *Alternaria* species causing diseases in tomato have been attended for their genetic diversity and ability to produce various toxic compounds that can be retrieved in tomatoes and tomato-derived products. *Alternaria solani* and *A. allternata* have been historically associated to early blight and brown spot, respectively. However, the concurrent presence of both pathogens on the same plant and the confusing symptomatology of early blight/brown spot at their initial stages led to the conception of disease complex (Vandecasteele et al., 2018). For instance, a survey by Kokaeva et al. (2018) showed that *A. alternata* and *A. solani* were frequently isolated in Russia, and their simultaneous presence was predominant in tomato samples hosting more than one species. Furthermore, novel diagnosis methods based on molecular analyses allowed the identification of other *Alternaria* species associated to tomato, which can be involved in the disease complex. For example, studies conducted in Italy revealed that *A. tenuissima* was present in 25% and 20% of symptomatic tomato samples collected in northern and southern regions, respectively, while the remaining part was identified as *A. alternata* (Bertuzzi et al., 2021; Sanzani et al., 2019). In similar research carried out in northwestern Algeria, species belonging to the phylogenetic section ‘alternaria’ represented more than 80% of isolates from tomato samples, with *A. tenuissima* being the dominant species (Bessadat et al., 2017).

Since the early stages of epidemics, *Alternaria* species may produce mycotoxins that accumulate within plant tissues and can contaminate fruits (Lou et al., 2013). Certain species are able to produce several mycotoxins belonging to different structural groups, while others are able to produce only few or single metabolites (Logrieco et al., 2009; Lou et al., 2013; Pose et al., 2010). The most frequently produced toxins include dibenzopyrone derivates such as alternariol (AOH) and alternariol monomethyl ether (AME), and tetramic acid derivatives like tenuazonic acid (TeA) (Pose et al., 2010). Although the role of these metabolites is not fully understood, they are probably involved in the infection process of *Alternaria* species. AOH and AME are pathogenicity factors supporting tomato tissue colonization and causing necrosis development, likely involved in mitochondrial apoptosis signaling (Wenderoth et al., 2019). TeA is a phytotoxic, non-host-specific compound likely linked to inhibition of the photosystem II (Meena et al., 2016). Surveys conducted in several countries in Europe and South America reported that TeA and AOH are primary contaminants in tomato retails, with detection frequencies up to 100% and concentrations from a few ng/kg to thousand *µ*g/kg (Ackermann et al., 2011; Bertuzzi et al., 2021; da Motta and Valente Soares, 2001; Noser et al., 2011; Terminiello et al., 2006). AOH, AME, and TeA are well-known to have mutagenic, estrogenic, and clastogenic effects in microbial and mammalian cell systems health (EFSA Panel on Contaminants in the Food Chain (CONTAM), 2011, 2016). Such a danger posed to human health triggered the European Union to deliver Recommendation (EU) 2022/553 that sets the limits for *Alternaria* toxins in processed tomatoes, which are equal to 5, 10, and 500 µg/kg for AOH, AME, and TeA, respectively.

Despite the substantial body of modeling work available in the literature, current models fail to represent the biological and ecological complexity of *Alternaria* diseases in tomato and the risk of mycotoxin contamination. Existing approaches predominantly target *A. solani* and were developed either to predict infection periods or to simulate disease progress, without addressing toxin production. With the notable exception of Waggoner and Horsfall (1969), who proposed a mechanistic framework, most models rely on empirical approaches (including statistical and machine-learning methods). These models generally represent isolated components of the pathogen life cycle (primarily infection (Madden et al., 1978; Srivastava and Gillespie, 1989; Waggoner and Horsfall, 1969) or reduce disease development to a weather-driven process, disregarding the underlying fungal biology (Gupta et al., 2020; Paul et al., 2019; Peña et al., 2012; Ruth et al., 2016; Saha and Das, 2014a, 2015b, a, b; Tadelo et al., 2022). Such simplifications constrain their explanatory power and limit their capacity to support integrated disease management strategies. Although systems such as FAST (Madden et al., 1978) and its derivatives (e.g., TOMCAST, CU-CAST, NJ-CAST) (Cowgill et al., 2005; Tietjen et al., 2001; Louws et al., 1996) have been successfully implemented to optimize fungicide applications against *Alternaria* infections on foliage and fruit, they provide no guidance for mitigating mycotoxin contamination. Likewise, the mechanistic model EPIDEM (Waggoner and Horsfall, 1969), despite incorporating key biological parameters of *A. solani*, does not account for mycotoxin production. Consequently, a critical component of the risk posed by *Alternaria* spp. (i.e., its toxicological dimension) remains unmodeled.

Given the knowledge recently gained on *Alternaria* species associated to diseases in tomato and safety threats due to mycotoxins, the current work proposes a mechanistic, weather-driven model to predict infection periods, disease progress, and risk of toxin contamination (specifically AOH, AME, and TeA). The model, which is called ALT-tomato, is based on key biological processes involved in epidemic development: i) production of conidia from overwintering sources ii) conidial dispersal, iii) infection by conidia, iv) latency period and secondary inoculum production, and v) production and accumulation of toxins within tomato fruit tissues. The model is parametrized for three *Alternaria* species primarily associated to tomato, i.e., *A. alternata*, *A. solani*, and *A. tenuissima*, and comprises their possible co-occurrence in epidemic development.

## 2 Materials and methods

### 2.1 Literature search

To develop the conceptual and mathematical structure of the model, the available information in the literature was used. The recent study by Salotti et al. (2024) was exploited as a basis, since the authors reviewed available information on the biology, ecology and epidemiology of *Alternaria* spp. affecting tomato, with a focus on ground information needed for the development of predictive models. A further search was carried out in July 2025 to review article of potential interest released after 2022 (i.e., the year in which the literature search was carried out by Salotti et al. (2024)). The new search was conducted using the same keyword proposed by Salotti et al. (2024) in three bibliographical databases: Scopus (https://www.scopus.com/ accessed on July 25), Web of Science (https://www.webofscience.com/ accessed on July 25), and CAB Abstracts (https://www.cabidigitallibrary.org/product/ca accessed on July 25). Articles were reviewed and selected; data on the influence of environmental conditions on *Alternaria*-tomato pathosystem were obtained directly from text, tables, or graphs; the software GetData Graph Digitizer 2.24 (http://getdata-graph-digitizer.com accessed on 5 May 2021) was used to obtain precise data from graphs.

### 2.2 Conceptualization of the system

The model was conceptualized based on common attributes of the life cycle of *Alternaria* species associated with tomato, with particular reference to three major species causing epidemics on foliage and fruits, i.e., *A. alternata*, *A. tenuissima*, and *A. solani*. Although the parasexual and sexual recombination have been supposed to occur based on genetic data in both large- and small-spored *Alternaria* species, sexual stages were not observed (Meng et al. (2015)). Therefore, the model considers the asexual reproduction as the most important stage for disease development, and the asexual conidia the basic unit able to cause infections. Overwintering conidia and mycelial fragments in infected debris and soil were considered as primary inoculum sources, as well as diseased volunteer plants or other solanaceous hosts. *Alternaria* conidia are produced from primary inoculum sources under favorable conditions of temperature (T from 10 to 30°C) and relative humidity (RH ≥ 65%) for the entire cropping period. Conidial dispersal follows a diurnal periodicity; thus, it is assumed that the amount of produced and dispersed conidia coincides. Conidia on susceptible tissues are able to germinate and directly penetrate the cuticle and epidermal cell walls with suitable T (from 5 to 35°C) and duration of leaf wetness (WD ≥ 3 hours). Penetration may also occur through stomata and wounds. Tomato susceptibility to *Alternaria* spp. infections is age-related, with an increase in susceptibility with foliage aging and fruit ripeness. Generally, symptoms become visible after 5 to 7 days on foliage and after 2 to 10 days on fruits, depending on their ripeness and incubation temperature, and secondary inoculum is produced on lesions. After infection, toxins are produced by *Alternaria* spp. mainly depending on T (with optimum 20 – 30°C) and accumulate within tissues over time. The system considers the production of TeA, AOH, and AME, as they have been commonly detected in fresh tomatoes and tomato-based products.

### 2.3 Model structure

**Figure 1:**
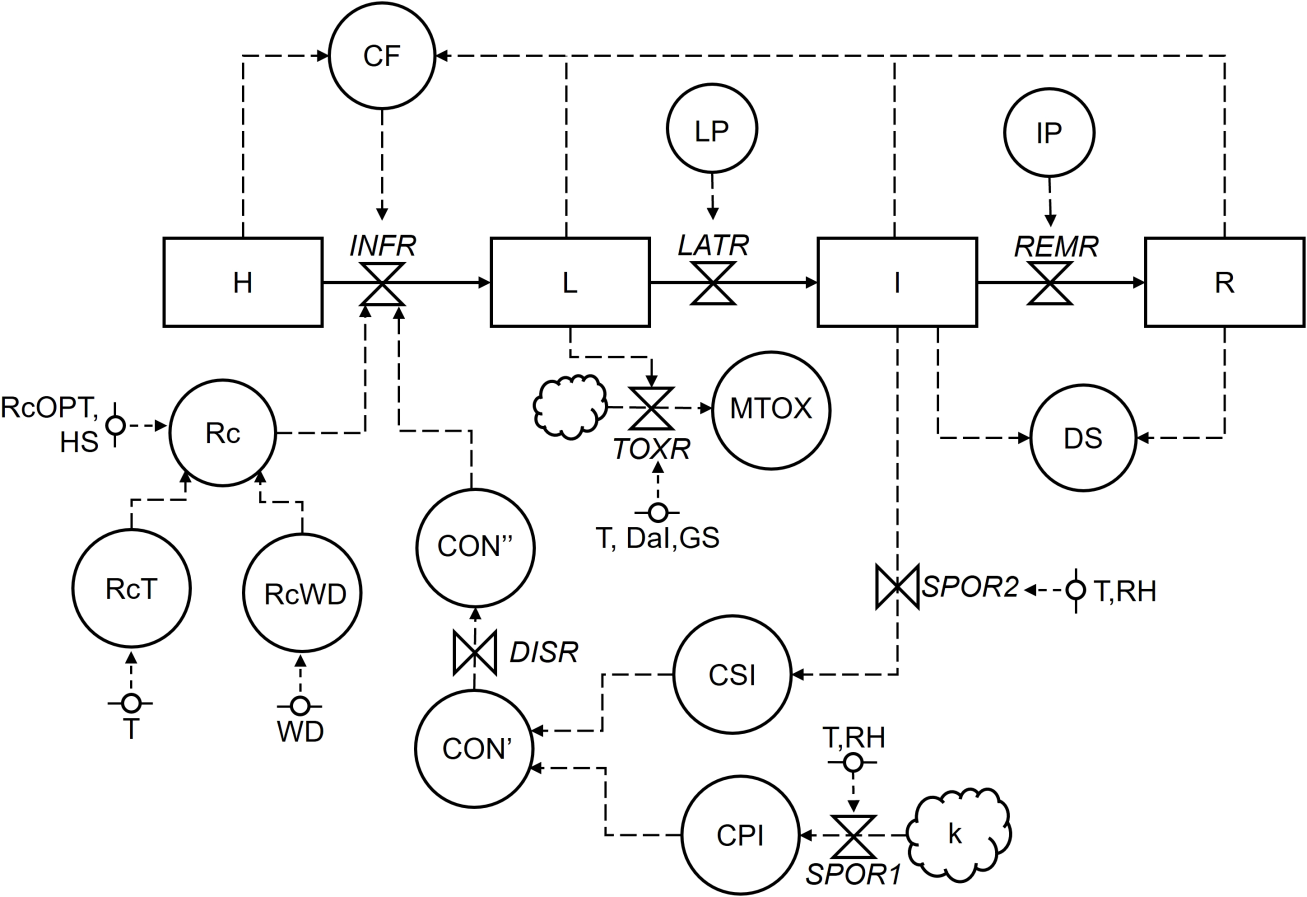
Relational diagram of the processes leading to infection, disease development, and mycotoxin contamination by *Alternaria* species in tomato. Acronyms for state variables, rates, and parameters are listed in **Table 2**. The core structure of the model is plant-focused, with sites evolving from healthy (H), to latent (L), to infectious (I), and finally to removed (R).

Following system analysis principles, literature information was analyzed and organized into a relational diagram (Leffelaar and Ferrari, 1989). The model core is based on the plant-focused structure proposed by (Zadoks, 1971), which considers the crop as large but finite number of sites. A site is defined as a fraction of host tissue that can be infected and develop into a lesion; sites have equal size and equal probability of becoming infected. During the epidemic, sites may assume the following, mutually exclusive stages: i) healthy; ii) latent – i.e., infected without visible, sporulating lesions; iii) infectious – i.e., infected with fertile, sporulating lesions); iv) removed – i.e., infected with old, no-longer sporulating lesions. Plant site stages represent the state variables of the model (boxes in **Figure 1**), while pathogen features and the environment are comprised as auxiliary (circles) and external, independent (segmented circles) variables. Specifically, the external variables include plant age (in weeks after transplanting) and host growth stages (in BBCH scale; Feller et al., 1995) as well as the weather variables, i.e., temperature (T, in°C), relative humidity (RH, in %), and duration of wetness (WD, in hours). The flux of sites (solid arrows) from one stage to the following one is regulated by rates (valves), which in turn are regulated by auxiliary and external variables through rules and mathematical equations (dotted arrows). Source variables (clouds) were included to account for primary inoculum sources that could not be quantified within model runs.

### 2.4 Mathematical framework

Mathematical equations were developed and parametrized using literature knowledge. The effect of the external and auxiliary variables on rates was drawn by pooling together quantitative data extracted from published experiments. Because the experiments used different fungal isolates, plant materials, measurement unites to express results, etc., data were rescaled in the 0 to 1 range. Rescaling was performed for data of each experiment by diving each value by the maximum value of the experiment. Rescaled data were then regressed against the influencing variable using different nonlinear regression equations. The equations were compared, and the one that provided the performances listed below was considered the most likely to be correct: i) the smallest value of Akaike’s Information Criterion (AIC) (Brunham and Anderson, 2002); ii) small standard errors for the estimates of parameters; iii) proper goodness-of-fit indexes, i.e., high R^2^ adjusted for the degree of freedom and Concordance Correlation Coefficient (CCC), and low Root Mean Square Error (RMSE) and Coefficient of Residual Mass (CRM). The software R (version 4.3.2; R Core Team. R: A Language and Environment for Statistical Computing. 2019; available at https://www.r-project.org/) was used for computations. The estimates of equation parameters were obtained using the non-linear regression *nls* function from “stats” package, which was applied to rescaled data versus the independent variable. The adjusted R^2^ was estimated by conducting a linear regression with the *lm* function of “stats” package between observed (collected) data and values predicted by the equation. The CCC, which indicates the difference between the best fitting line and the perfect agreement line (Lin, 1989), was estimated with the *CCC* function of “DescTools” package. The RMSE, which represents the average distance between the observed data and the fitted line (Nash and Sutcliffe, 1970), was calculated with the *rmse* function of “modeler” package. The CRM, which indicates the tendency of the fitting line to over- or underestimate the observed data (negative or positive CRM values, respectively) (Nash and Sutcliffe, 1970), was directly calculated as the ratio between residuals of observed—estimated values and observed values.

### 2.5 Model validation in predicting *Alternaria* spp. epidemics

The ability of the model to describe *Alternaria* spp. epidemics in real, field conditions was evaluated using eight epidemics (disease progress curves) retrieved from literature. In all cases, the epidemics were originated from natural inoculum, the symptoms on plants were attributable to early blight or brown spot, and the disease severity was recorded on untreated plots of susceptible tomato varieties. Details on the epidemics are summarized in **Table 1**. In the validation process, it is assumed that each epidemic is triggered by a potential quantity of inoculum sources (represented by the parameter *k*; **Figure 1** and **Table 2**), which are responsible for the production of primary inoculum. Information to calculate k was not available in literature; therefore, the values of *k* used to initialize the model were estimated empirically by running the model with varying *k* values (in the 0 to 1 range) and selecting the one that allowed the closest agreement between predicted and observed final disease severity. The values of *k* are reported in **Table 1**. The disease progress predicted by the model (i.e., sum of infectious and removed sites) and the one observed in field were compared, and the goodness-of-fit was evaluated through CCC, RMSE, and CRM (calculated as described above: Section 2.4).

**Table 1:** Summary description of epidemics used for model validation.

Location	Year	Code	Final disease	*k*	Weather data
Ottawa, Ontario, Canada	1969	CA-69	73	0.11	Ottawa station (45°19’N, 75°40’W), 13 km
	1971	CA-71	92	0.21	
Bengalore, Karnataka, India	2012	KA-12	86	0.21	Bengalore station (13°12’N, 77°42’E), 30 km
	2013	KA-13	95	0.15	
	2014	KA-14	100	0.3	
	2015	KA-15	100	0.01	
	2016	KA-16	100	0.005	
Foggia, Apulia, Italy	2017	IT-17	26	0.33	Foggia station (41°26’N, 15°32’E), 10 km

**Table 2:** List of variables, rates, and parameters used in the model, and their units.

Acronym	Description	Unit
State variables		
H	Healthy sites	0 to 1
I	Infectious sites, where visible lesions produce conidia	0 to 1
L	Latent sites, where disease symptoms are not visible	0 to 1
R	Removed sites, where visible lesions are old and no more sporulating	0 to 1
Rates		
DISR	Rate of dispersal of conidia	0 to 1
INFR	Rate of infection by conidia	0 to 1
LATR	Rate of latency	0 to 1
REMR	Rate of removal	0 to 1
SPOR1	Rate of conidia production on primary inoculum sources	0 to 1
SPOR2	Rate of conidia production on secondary inoculum sources	0 to 1
TOXR	Rate of toxin production	0 to 1
Parameters		
*a* to *h*, *l*, *m*	Parameters of Equations 3, 5, 10, 11, 14	N
*ip*	Duration of infectious period in optimum conditions	N days
*k*	Abundance of primary inoculum sources	0 to 1
*lp*	Duration of latency period in optimum conditions	N days
*r*	Apparent infection rate	N
RcOPT	Optimum Rc value	N
Auxiliary and computed variables		
CF	Correction factor for diseased sites	0 to 1
CON’	Abundance of conidia on inoculum sources	N
CON”	Abundance of conidia on host tissues	N
CPI	Abundance of conidia on primary inoculum sources	N
CSI	Abundance of conidia on secondary inoculum sources	N
DS	Disease severity	0 to 1
IP	Infectious period	N
LP	Latency period	N
MTOX	Mycotoxin contamination	N
Rc	Basic infection rate corrected for the removal	N
External variable		
HS	Susceptibility of the host depending on host age	0/1
GS	Susceptibility of the host depending on host growth stage	0/1
RH	Relative humidity	%
T	Temperature	°C
WD	Duration of wetness	N hours
Driving functions		
*f(DaI)*	Equation for the effect of time on toxin production	0 to 1
*f(RH)*	Equation for the effect of relative humidity on sporulation	0 to 1
*f(T)*	Equation for the effect of temperature on sporulation or toxin production	0 to 1
RcT	Rc modifier for temperature	0 to 1
RcWD	Rc modifier for wetness duration	0 to 1

Units as 0 to 1 refer to a dimensionless, continuous variable; units as 0/1 refer to a dimensionless, binomial variable.

## 3 Results

### 3.1 Model description

The relational diagram showing the relationships of variables involved in the development of *Alternaria* spp. epidemic on tomato is reported in 1, with acronyms explained in **Table 2**. The core of the model is based on host sites that during an epidemic can assume one of the following conditions (that are mutually exclusive): i) healthy (H); ii) latent (L), i.e., infected but not symptomatic; iii) infectious (I), i.e., with lesions producing spores; and iv) removed (R), i.e., old and non-sporulating lesions removed from the system. Sites become infected (i.e., transit from H to L) at an infection rate (INFR), which depends on the abundance of conidia on host tissues. Conidia are produced on a *k* primary inoculum sources (CPI) and on I sites (CSI) at sporulation rates (SPOR1 and SPOR2, respectively) that depends on T and RH. Conidia produced on both sources accumulate in CON’ and then are daily dispersed (CON”) at a dispersal rate (DISR). The INFR also depends on a correction factor (CF) for diseased sites (i.e., L, I, R) and a multiplication factor representing the corrected basic infection rate (Rc), which in turn depends to an optimum basic infection rate (RcOPT) and its modifiers for T (RcT) and WD (RcWD) that are *Alternaria* species-specific. Infections occur during a period of host susceptibility (HS) that depends by host plant age and growth stage. L sites become I sites at a latency rate (LATR) that depends on a latency period (LP). I sites are then removed from the system, becoming R sites, at a removal rate (REMR) that depends on an infectious period (IP). The ability of *Alternaria* spp. to produce toxins after infection is accounted for each infection event. Toxins are produced according to the mycotoxin production rate (TOXR) that depends on T and time (expressed as days after infection, DaI), and accumulate into the variable MTOX over time. For the sake of simplicity, the model does not incorporate plant host processes as growth and senescence, as well as disease aggregation and lesion expansion, which may cause disease progress even if new infections do not occur (Loomis and Adams, 1983). The model operates from tomato transplanting to harvest at hourly timestep to better account for daily fluctuations in T, RH, and WD, with the only exception of TOXR that is calculated at daily timestep. At the beginning of model runs, H is set equal to 1 representing a crop that is entirely healthy, and flows from one stage to the following one are calculated as a proportion of H, i.e., in a 0–1 scale.

### 3.2 Production of conidia from primary inoculum sources

Conidia are produced on *k* primary inoculum sources (CPI) at a sporulation rate (SPOR1) that depends on T. On any i*^th^* hour, the contribution of these conidia to CON’ is calculated by Equation 1 as follows:

(1) CPI=k×SPOR1

where *k* represents the abundance of primary inoculum sources and assumes values from 0 to 1; and SPOR1 is a function of T and RH, so that

(2) SPOR1=f(T)×f(RH)

The f(T) in Equation 2 is calculated by a BETE equation (Analytis, 1977) in the following from:

(3) $$f(T)={\left(a\times Teq^{b}\times (1-Teq)\right)}^{c}$$

where *a*, *b*, and *c* are equation parameters; and Teq represents the equivalent of T (in°C) calculated based on minimum (Tmin) and maximum (Tmax) temperatures as follows:

(4) Teq=(T−Tmin)/(Tmax−Tmin)

To develop and parametrized Equations 3, 4 for *A. alternata*, data by Pearson and Hall (1975) and Bessadat et al. (2014) are used. Cardinal temperatures, estimates of equation parameters and their standard error are Tmin = 3°C, Tmax = 40°C, *a* = 4.097 ± 0.150, *b* = 1.032, *c* = 3.669 ± 0.660, with adjusted R^2^ = 0.739, CCC = 0.85, RMSE = 0.194, and CRM = –0.02. For *A. solani*, data by Naik et al. (2010); Singh (1967); Waggoner and Horsfall (1969); Sinha and Alam (2017), and Somappa et al. (2013) are used, resulting in Tmin = 5°C, Tmax = 32°C, *a* = 5.068 ± 0.197, *b* = 1.383 ± 0.066, *c* = 3.511 ± 0.681, with adjusted R^2^ = 0.803, CCC = 0.899, RMSE = 0.159, and CRM = 0.005. For *A. temuissima*, data by Bessadat et al. (2014) and Salotti et al. (2025a) are used, resulting in Tmin = 0°C, Tmax = 40°C, *a* = 5.041 ± 0.545, *b* = 1.372 ± 0.162, *c* = 1.358 ± 0.353, with adjusted R^2^ = 0.908, CCC = 0.959, RMSE = 0.102, and CRM = 0.001.

The f(RH) in Equation 2 is calculated by an asymptotic equation as follows:

(5) $$f(RH)=1-d^{\left(RH-RHmin\right)}$$

where *d* is the equation parameter; and RHmin represents the minimum value of RH (in %) that support conidia production, i.e., RHmin = 65 based on results by Naik et al. (2010). Equation 5 was developed and parametrized using data by Naik et al. (2010) and Somappa et al. (2013) which refer to *A. solani*. The estimate and standard error of *d* were 0.874 ± 0.011, with adjusted R^2^ = 0.987, CCC = 0.995, RMSE = 0.038, and CRM = –0.001. Because no data on the effect of RH on sporulation were retrieved for *A. alternata* and *A. tenuissima*, Equation 5 was used for all the species. CPI accumulates in the variable CON’, together with CSI (described in Section 3.5), so that CON’ = CPI+ CSI.

### 3.3 Dispersal of conidia

The dispersal of air-borne *Alternaria* conidia is positively correlated with T and negatively correlated with RH, leading to a diurnal periodicity with a peak in the afternoon hours (Gottwald et al., 1997; Jambhulkar et al., 2016; Pearson and Hall, 1975). The model assumes that all conidia in CON’ are dispersed, so that DISR is set equal to 1 and the potential amount of conidia deposited on plant surfaces is CON” = CON’.

### 3.4 Infection by conidia

Conidia on plant surfaces can cause infection at the infection rate (INFR) that governs the transit of sites from H to L. The INFR is the product Equation 6 as follows:

(6) INFR=Rc×CF×CON'×HS

where Rc represents the corrected basic infection rate (Van der Plank, 1963), i.e., the proportion of daughter lesions generated per mother lesion; CF is correction factor diseased sites; and HS defines periods of plant susceptibility to infection. The HS value depends on plant age expressed as week after tomato transplanting. Specifically, HS = 1 starting from five weeks after transplanting (Horsfall and Heuberger, 1942), otherwise HS = 0.

The CF accounts for diseased sites (i.e., L, I, and R) that cannot be infected anymore, and is calculated by Equation 7 as follows:

(7) CF=1−(L+I+R)/(H+L+I+R)

The Rc depends on an optimum corrected basic infection rate (RcOPT, i.e., the basic infection rate under optimal environmental conditions for epidemic development on a susceptible host) and on modifiers (Loomis and Adams, 1983) for the effect of temperature (RcT) and duration of wetness (RcWD) that are *Alternaria* species-specific. Therefore, Rc is calculated for each species as follows by Equation 8:

(8) Rc=RcOPT×RcT×RcWD

According to Sun and Zeng (1994), the value of RcOPT is estimated depending on the apparent infection rate (*r*), and the latency (*lp*) and infectious (*ip*) under optimal conditions:

(9) RcOPT=r/(exp(−r×lp)−exp(−r×(lp+ip)))

The *r* value in Equation 9 is retrieved from Pandey et al. (2003) as the average of *r* values reported for susceptible tomato cultivars under natural epidemic conditions in unprotected crops; thus, *r* = 0.095. Because *A. alternata*, *A. solani*, and *A. tenuissima* have similar requirements in symptoms development (Salotti et al., 2024), the values of *lp* and *ip* are set equal to 7 and 30, irrespectively from the species (Kemmitt, 2002; Waggoner and Horsfall, 1969). Therefore, the value implemented in the model is RcOPT = 0.2. The modifier RcT defines the effect of T on infection and the temperature-dependent equation is developed and parametrized for each species. For *A. alternata*, RcT is drawn using the BETE equation (Analytis, 1977) in the form of Equation 3 using data by Malathrakis (1983), with *a* = 5.251 ± 0.237, *b* = 1.452 ± 0.071, and *c* = 1.929 ± 0.237, and goodness-of-fit of adjusted R^2^ = 0.981, CCC = 0.992, RMSE = 0.051, and CRM = –0.002. For *A. solani*, and *A. tenuissima*, RcT is developed based on Briere et al. (1999) temperature-dependent equation in the following form:

(10) $$RcT=e\times T\times (T-Tmin)\times {\left(\sqrt{Tmax-T}\right)}^{\left(1/f\right)}$$

where *e* and *f* are equation parameters; Tmin and Tmax are cardinal temperatures, i.e., minimum and maximum temperature that support infection, respectively. To develop and parametrized Equation 10 for *A. solani*, data by Chohan et al. (2015); Kemmitt (2002), and Nightingale and Ramsey (1936) are exploited. Cardinal temperatures, estimates of equation parameters and their standard error are Tmin = 0°C, Tmax = 40°C, *e* = 0.0002 ± 0.0001, *f* = 0.672 ± 0.113, with adjusted R^2^ = 0.754, CCC = 0.875, RMSE = 0.143, and CRM = 0.047. For *A. tenuissima*, data by Salotti et al. (2025a) are used, resulting in Tmin = 5°C, Tmax = 35°C, *e* = 0.0009 ± 0.0002, *f* = 1.955 ± 0.502, with adjusted R^2^ = 0.905, CCC = 0.954, RMSE = 0.102, and CRM = 0.035. The RcWD expresses the effect of WD on infection. Similarly to RcT, RcWD is designed for each species using a Gompertz equation in the following form:

(11) RcWD=exp(−g×exp(−h×WD))

where *g* and *h* are equation parameters. For *A. solani*, data by Waggoner and Horsfall (1969) and Vloutoglou and Kalogerakis (2000) are exploited for parametrization, resulting in *g* = 89.47 ± 2.57, *h* = 0.640 ± 0.039, with adjusted R^2^ = 0.998, CCC = 0.999, RMSE = 0.019, and CRM = –0.001. For *A. tenuissima*, data of Salotti et al. (2025a) are used, resulting in *g* = 4.919 ± 0.833, *h* = 0.324 ± 0.031, with adjusted R^2^ = 0.994, CCC = 0.998, RMSE = 0.023, and CRM = –0.008. Because no literature information was available concerning the effect of WD on *A. alternata* infection, available data on the effect of WD on germination were compared for the three species (Pearson and Hall, 1975; Malathrakis, 1983; Waggoner and Parlange, 1974b, a; Salotti et al., 2025a). *A. tenuissima* response to WD reveled high concordance (R^2^ = 0.999) with the response of *A. alternata*, therefore, the parametrization of *A. tenuissima* RcWD was selected as a proxy for *A. alternata* RcWD.

### 3.5 Latency period and secondary inoculum production

Sites transit from L to I at a latency rate (LATR) that depends on LP, i.e., the latency period. Fragmented information is available in literature concerning the onset of symptoms and pathogen evasion from host tissues. Therefore, a fixed period of 7 days is kept as representative of average LP on both leaves and fruits (Kemmitt, 2002; Malathrakis, 1983; Waggoner and Horsfall, 1969; Nightingale and Ramsey, 1936). Sites in I are then removed from the system and flow into R at a rate of removal (REMR), which depends on IP, i.e., the infectious period. *Alternaria* species are able to sporulate on various substrata in tomato fields other than lesions on leaves and fruits, including senescent and dead plant material Pearson and Hall (1975). The model assumes that sporulation is supported even on old lesions for the entire duration of the epidemic, therefore, REMR is set equal to 0. The I sites contribute to the production of secondary inoculum at a sporulation rate (SPOR2). Similarly to SPOR1, SPOR2 depends on T and RH as defined in Equations 2 to 5. The contribution of secondary conidia (CSI) to CON’ is calculated by Equation 12 as::

(12) CSI=I×SPOR2

### 3.6 Mycotoxin production

Toxins produced by *Alternaria* spp. can accumulate into tomato tissues after infection. The model focuses on the production and accumulation of AOH, AME, and TeA in tomato fruits. Therefore, the model accounts for toxin production and accumulation at a rate of toxin production (TOXR) that depends on T, tomato growth stage (GS), and days after infection (DaI). After every infection event, TOXR is calculated for each *Alternaria* species and toxin as follows:

(13) TOXR=f(T)×f'(DaI)×GS

The f(T) in Equation 13 is derived by Salotti et al. (2025b), which proposes BETE equations (Analytis, 1977) to describe the effect of T on the production of AOH, AME, and TeA, for *A. alternata*, *A. tenuissima*, and *A. solani*. For model calculation, T correspond to the average daily temperature (in °C). Concerning the effect of time (DaI) on TOXR, no species-specific knowledge is available in literature; the f(DaI) is then developed and parametrized for AOH, AME, and TeA and used for all species. The f’(DaI) was the first derivative of the f(DaI) Gompertz equation in the following form:

(14) f(DaI)=exp(−l×exp(−m×DaI))

where *l* and *m* are equation parameter; for every j*^th^* day in which an infection occurs, DaI is calculated starting as the cumulative number of days after the infection. For AOH, Equation 14 is designed exploiting data by Qin et al. (2022) and Pose et al. (2010), with *l* = 10.0 ± 1.52, *m* = 0.462 ± 0.201, adjusted R^2^ = 0.864, CCC = 0.933, RMSE = 0.153, CRM = –0.075. For AME, Equation 14 is designed using data by Qin et al. (2022) and Pose et al. (2010), with *l* = 9.5 ± 1.829, *m* = 0.464 ± 0.314, adjusted R^2^ = 0.822, CCC = 0.909, RMSE = 0.176, CRM = 0.007. For TeA, Equation 14 is designed using data by Qin et al. (2022); Sun et al. (2025), and Pose et al. (2010), with *l* = 8.294 ± 1.436, *m* = 0.254 ± 0.096, adjusted R^2^ = 0.741, CCC = 0.863, RMSE = 0.194, CRM = –0.006. The GS value depends on tomato growth stage, expressed in BBCH (Feller et al., 1995). Greenhouse and field studies discovered that fruit infection can occur even on unripe, green fruits, although they develop limited symptoms compared to ripe fruits (Nightingale and Ramsey, 1936; Pearson and Hall, 1975; Qaryouti et al., 2012). Therefore, the model assumes that GS = 1 starting from BBCH = 71 (i.e., the first fruit has reached typical size and form), otherwise GS = 0.

### 3.7 Predicted disease severity and mycotoxin contamination risk

**Figure 2:**
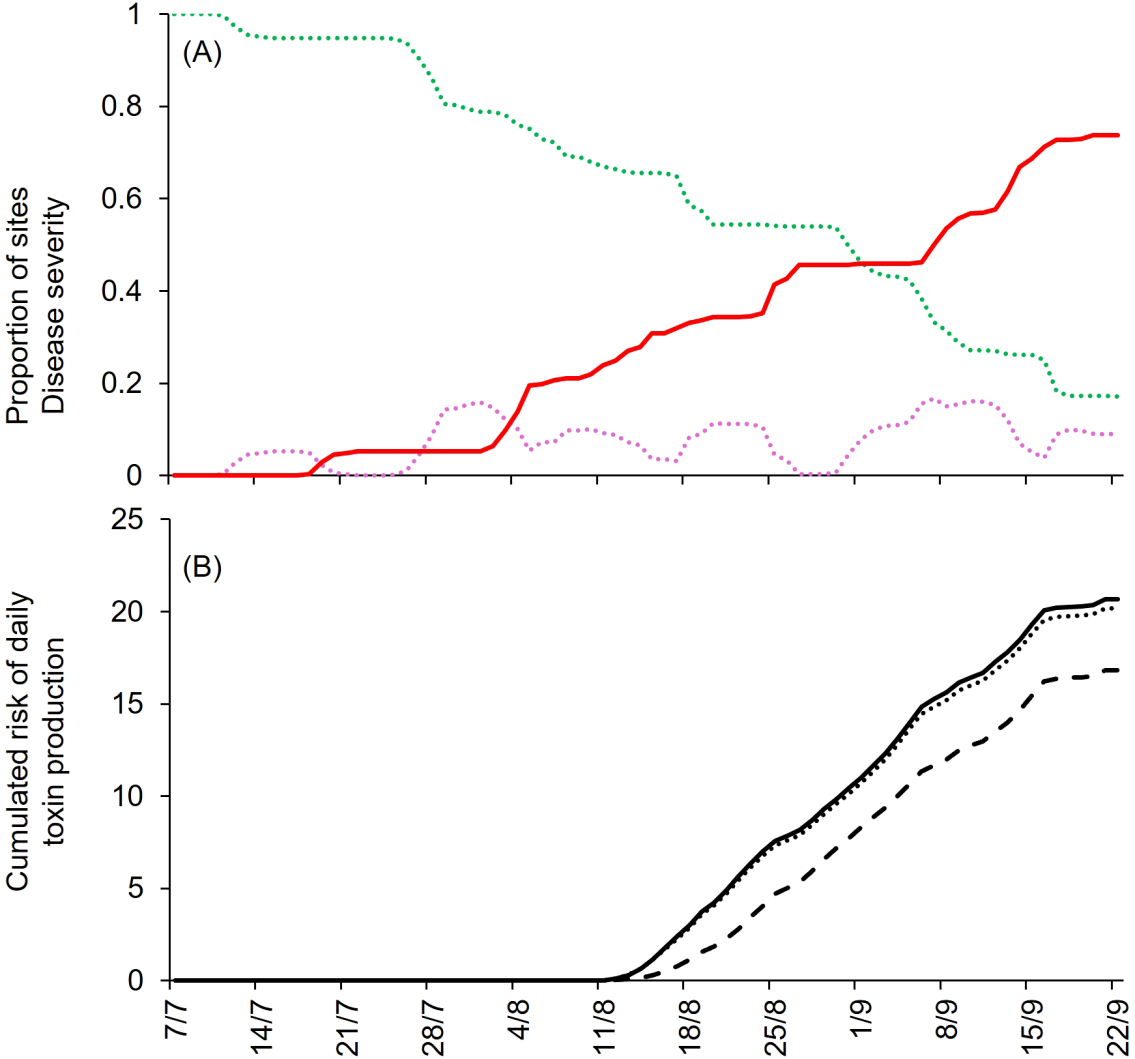
Model output for the tomato field in Ottawa, Ontario, Canada, in 1969 (CA-69); the model was run from July 7 to September 22. **(A)** Dynamics of sites: healthy (H, green dotted line), latent (L, purple dotted line), infectious (I, red dotted line), removed (R, gray dotted line), and disease severity (DS, red solid line). **(B)** Cumulated risk of daily production of mycotoxins: alternatiol (AOH; solid line), alternariol monomethyl ether (AME; dotted line), and tenuazonic acid (TeA; dashed line).

The model calculates the progress of disease severity (DS) during the epidemic as the sum of the proportion of sites that show symptoms, i.e., infectious and removed sites, so that DS = I + R. Finally, the model calculates the risk of mycotoxin contamination as the accumulation of AOH, AME, and TeA during the epidemic. Specifically, for each toxin, the daily TOXR values accumulate into the variable MTOX. An example of model output in presented in **Figure 2**, showing the dynamic of H, L, I, and R sites, and the increase of DS and MTOX over time.

### 3.8 Model validation in predicting *Alternaria* spp. epidemics

The ability of the model to describe *Alternaria* spp. epidemics in real, field conditions was evaluated using eight epidemics (**Table 1**) recorded on tomato foliage between 1969 and 2017 in Canada (Basu, 1974), India (Paul et al., 2019), and Italy (Delpero et al., 2018), with final disease severity on leaves ranging from 26 to 100%.

**Figure 3:**
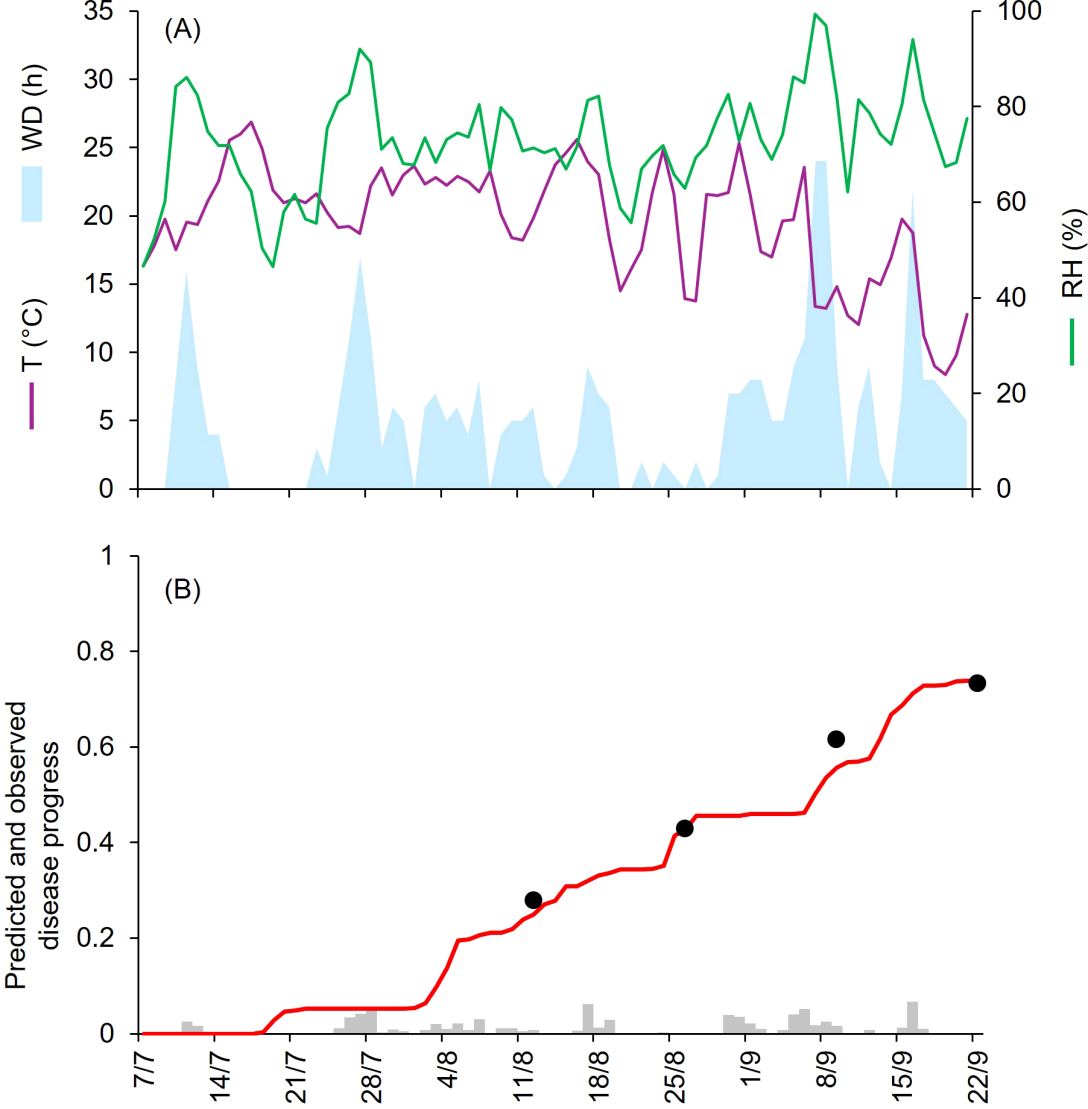
Predicted and observed disease progress at Ottawa, ON, Canada, in 1969 (CA-69). **(A)** Weather variables: air temperature (T, in °C; purple line), relative humidity (RH, in %; green line), and wetness duration (WD, in hours; light blue area). **(B)** Infection severity predicted by the model (light gray bars), disease severity predicted by the model (red line), and observed disease incidence (black dots).

**Figure 4:**
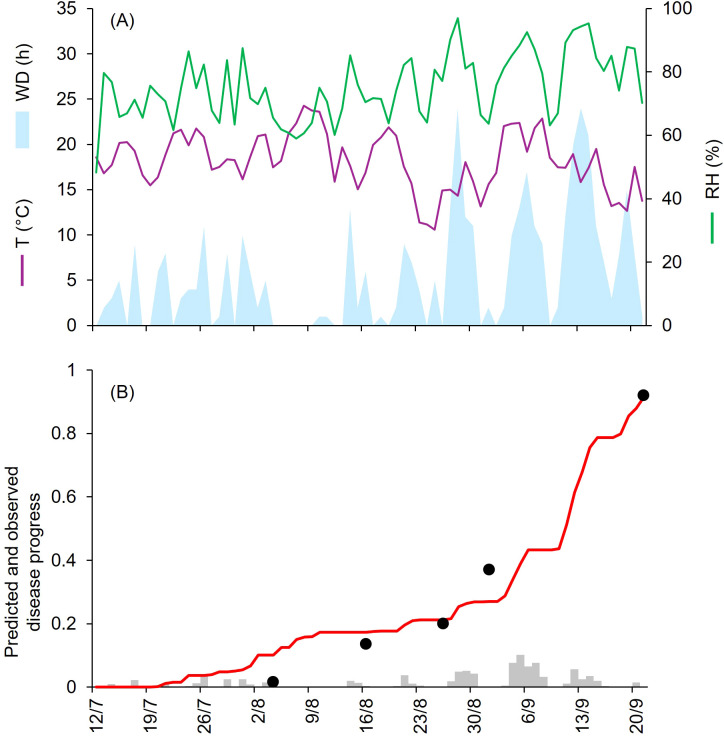
Predicted and observed disease progress at Ottawa, ON, Canada, in 1971 (CA-71). **(A)** Weather variables: air temperature (T, in °C; purple line), relative humidity (RH, in %; green line), and wetness duration (WD, in hours; light blue area). **(B)** Infection severity predicted by the model (light gray bars), disease severity predicted by the model (red line), and observed disease incidence (black dots).

At CA-69 and CA-71, epidemics developed between July and September, with final disease severity of 73% and 92%, respectively (**Table 1**). Both growing seasons were characterized by cool and humid weather. At CA-69 and CA-71, the daily average T, RH, and cumulated WD hours were 19.6°C, 72.6%, 392 h, and 18.1°C, 75.2%, and 394 h, respectively (**Figures 3A**, **4A**). At CA-69, infections were regularly predicted during the season, so that the predicted disease severity increased almost linearly over time (**Figure 3B**). For the goodness-of-fit of predicted versus observed data, concordance correlation coefficient (CCC) = 0.962 and root mean square error (RMSE) = 0.094. The model showed a tendency toward underestimation (coefficient of residual mass (CRM) = 0.093). At CA-71, the model predicted infections in two main windows, corresponding to periods with longer wetness duration. The first window occurred from mid-July and the beginning of August, while the second window extended from mid-August to mid-September, leading to a marked increase in predicted disease severity in the second half of the growing season (**Figure 4B**). Goodness-of-fit of predicted versus observed data had a CCC = 0.981 and a RMSE = 0.07. A CRM = 0.021 indicated slight underestimation by the model.

**Figure 5:**
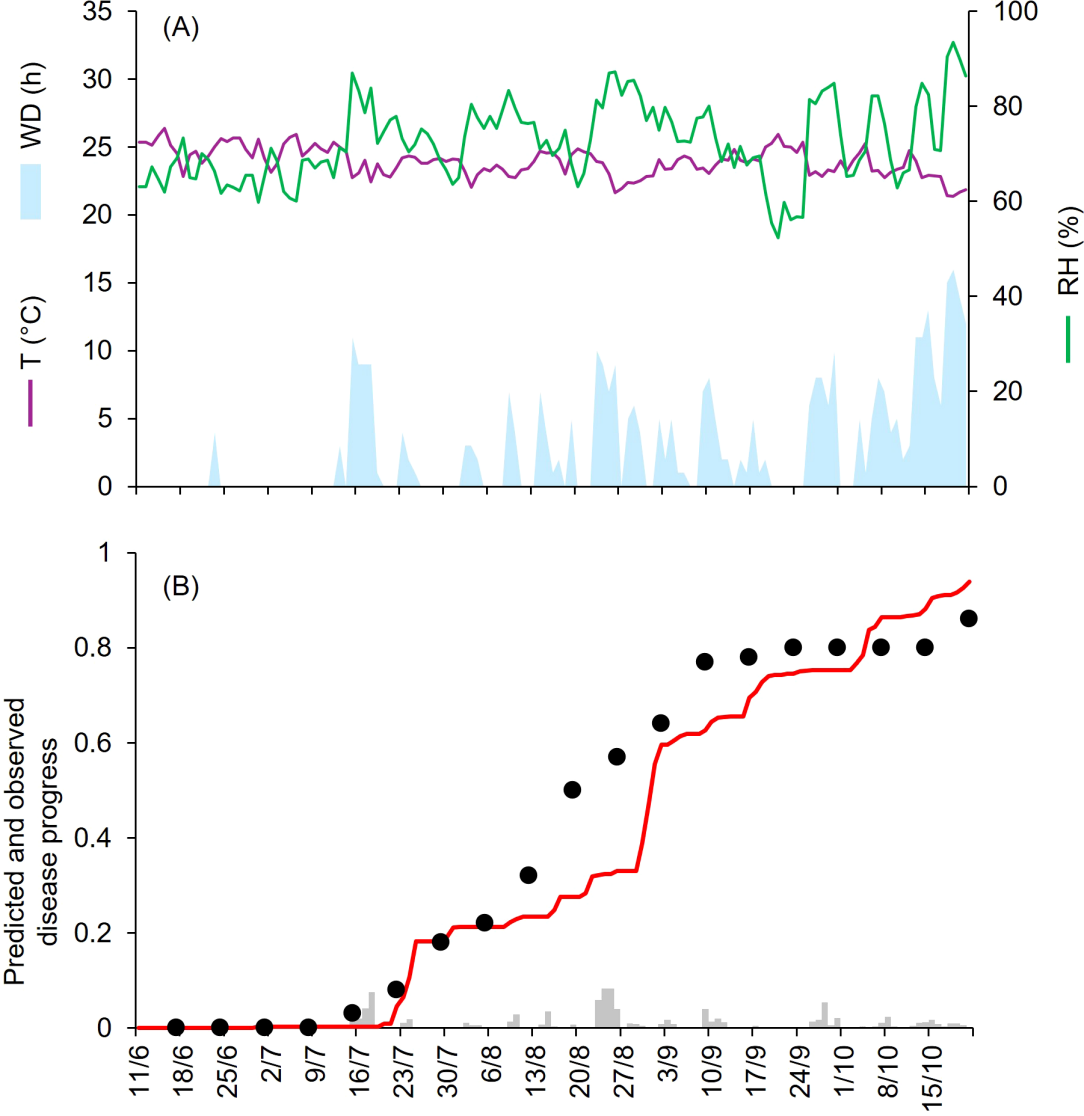
Predicted and observed disease progress at Bengalore, KA, India, in 2012 (KA-12). **(A)** Weather variables: air temperature (T, in °C; purple line), relative humidity (RH, in %; green line), and wetness duration (WD, in hours; light blue area). **(B)** Infection severity predicted by the model (light gray bars), disease severity predicted by the model (red line), and observed disease incidence (black dots).

**Figure 6:**
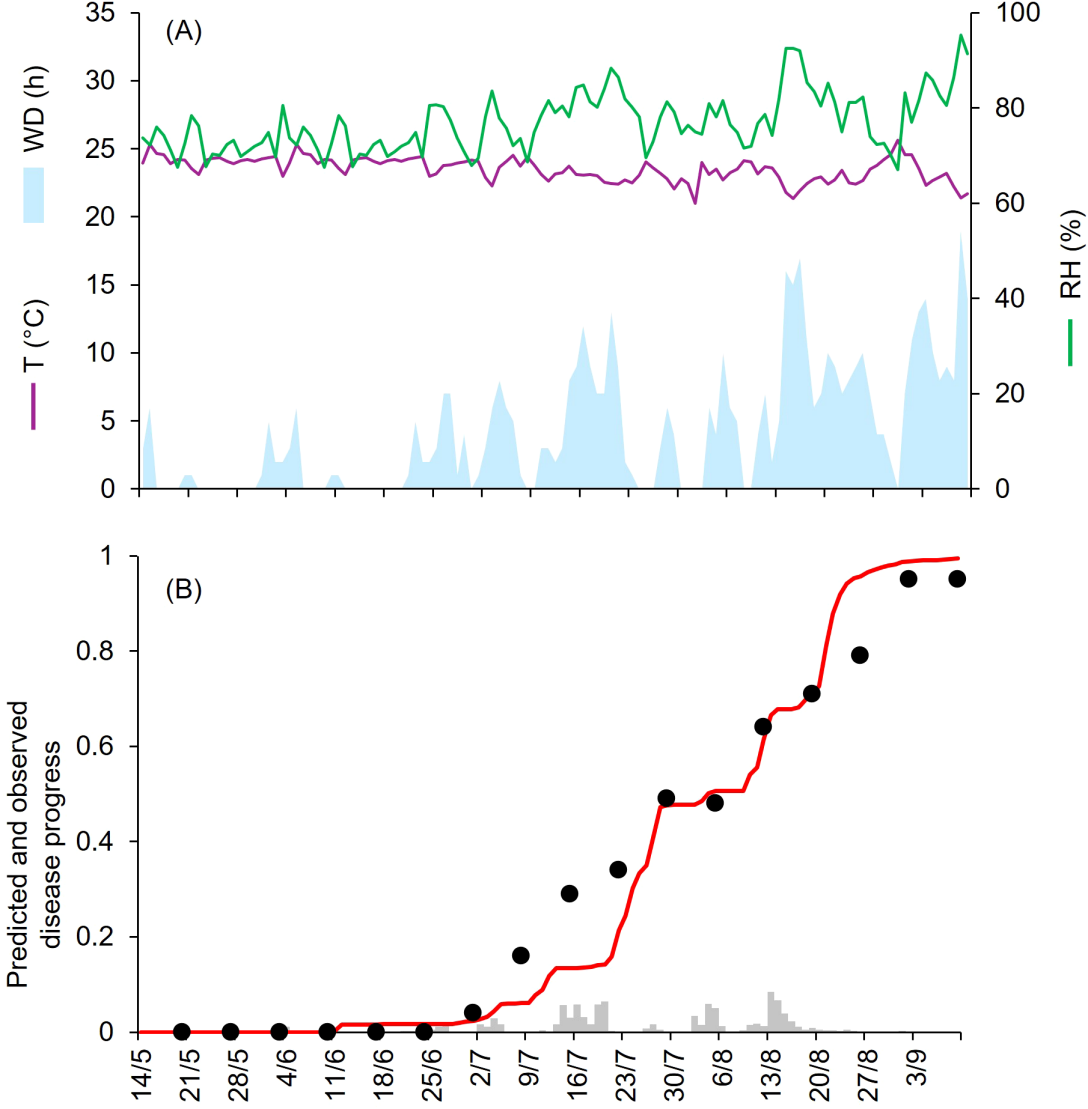
Predicted and observed disease progress at Bengalore, KA, India, in 2013 (KA-13). **(A)** Weather variables: air temperature (T, in °C; purple line), relative humidity (RH, in %; green line), and wetness duration (WD, in hours; light blue area). **(B)** Infection severity predicted by the model (light gray bars), disease severity predicted by the model (red line), and observed disease incidence (black dots).

**Figure 7:**
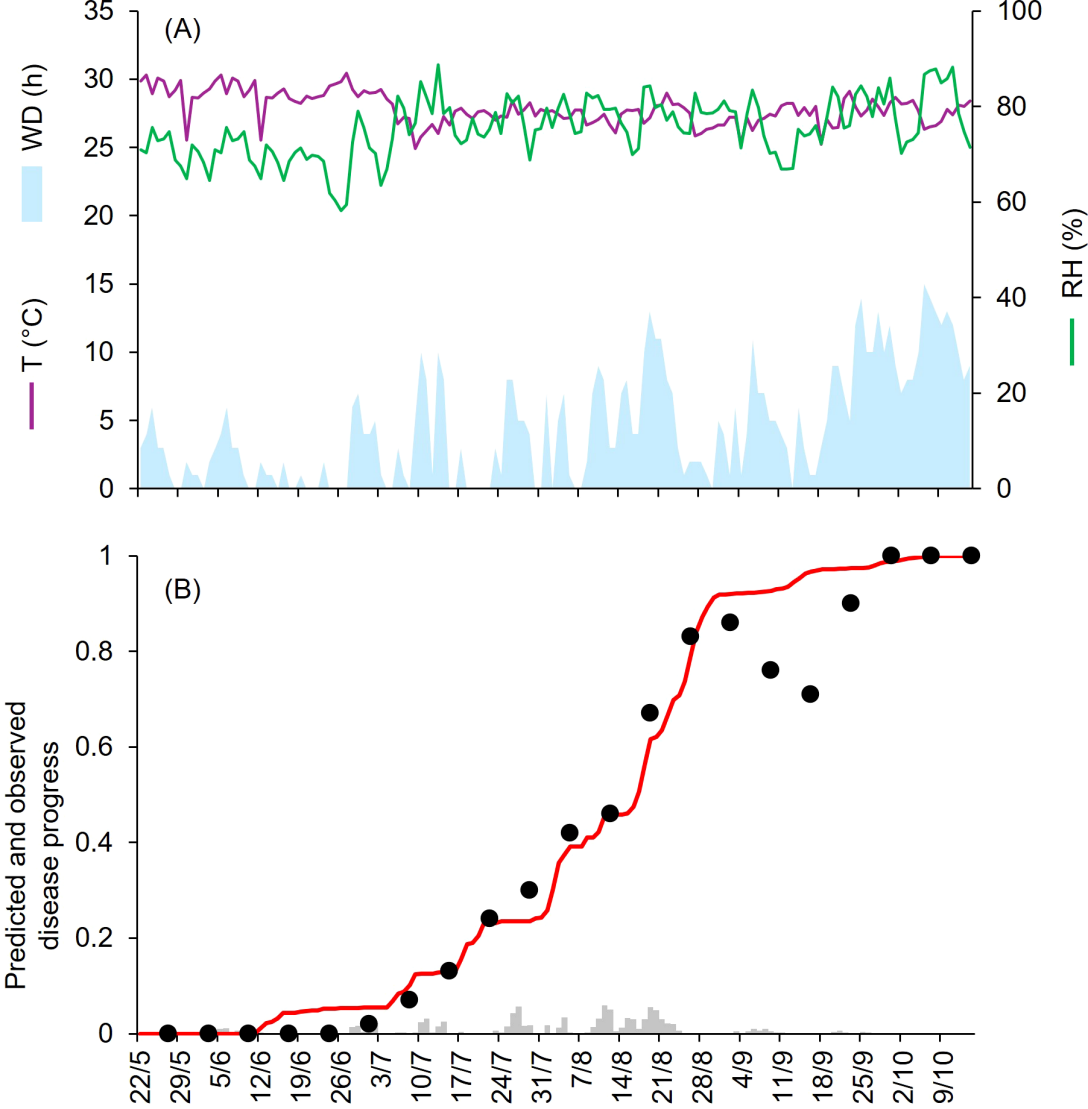
Predicted and observed disease progress at Bengalore, KA, India, in 2014 (KA-14). **(A)** Weather variables: air temperature (T, in °C; purple line), relative humidity (RH, in %; green line), and wetness duration (WD, in hours; light blue area). **(B)** Infection severity predicted by the model (light gray bars), disease severity predicted by the model (red line), and observed disease incidence (black dots).

**Figure 8:**
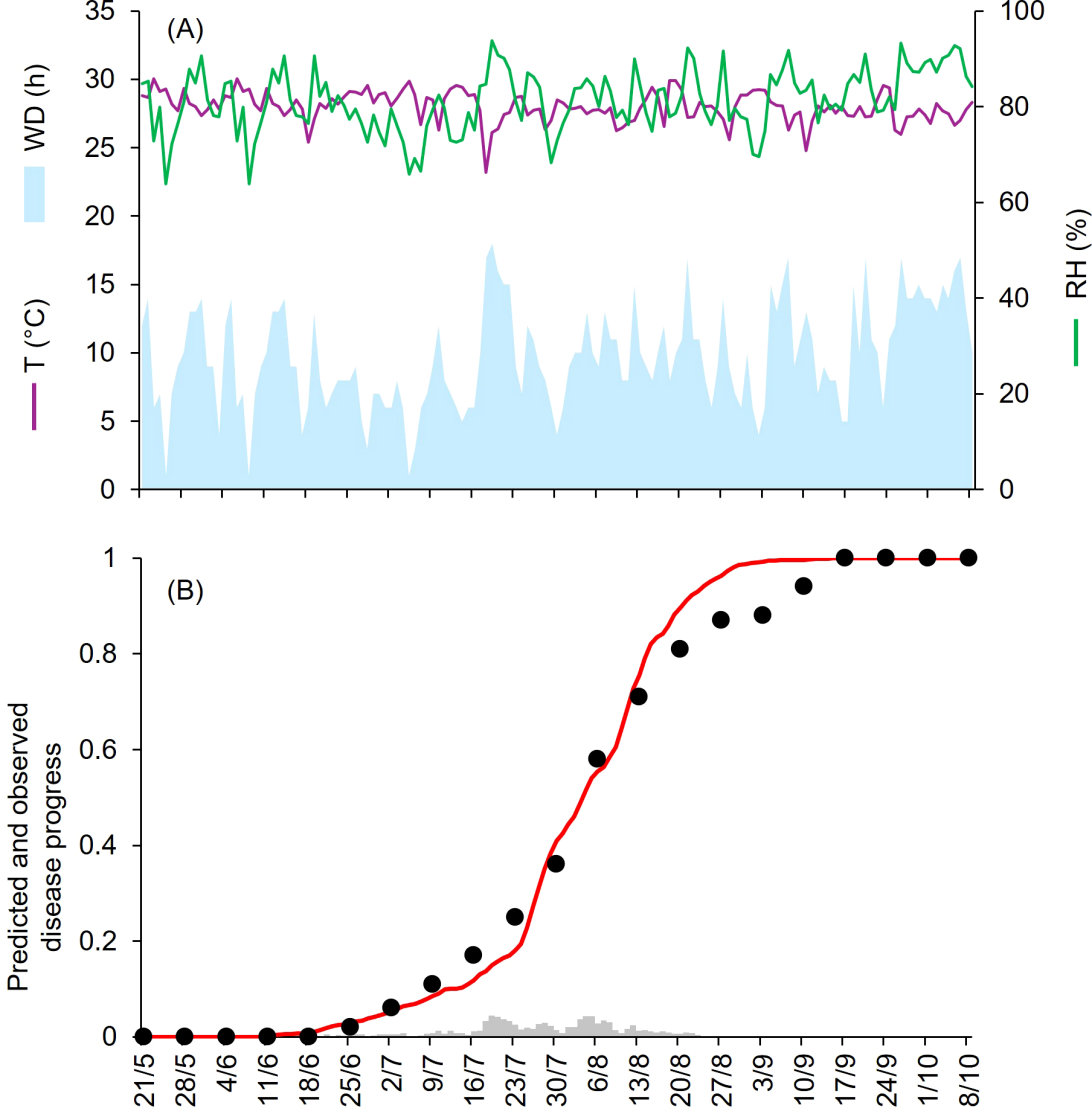
Predicted and observed disease progress at Bengalore, KA, India, in 2015 (KA-15). **(A)** Weather variables: air temperature (T, in °C; purple line), relative humidity (RH, in %; green line), and wetness duration (WD, in hours; light blue area). **(B)** Infection severity predicted by the model (light gray bars), disease severity predicted by the model (red line), and observed disease incidence (black dots).

**Figure 9:**
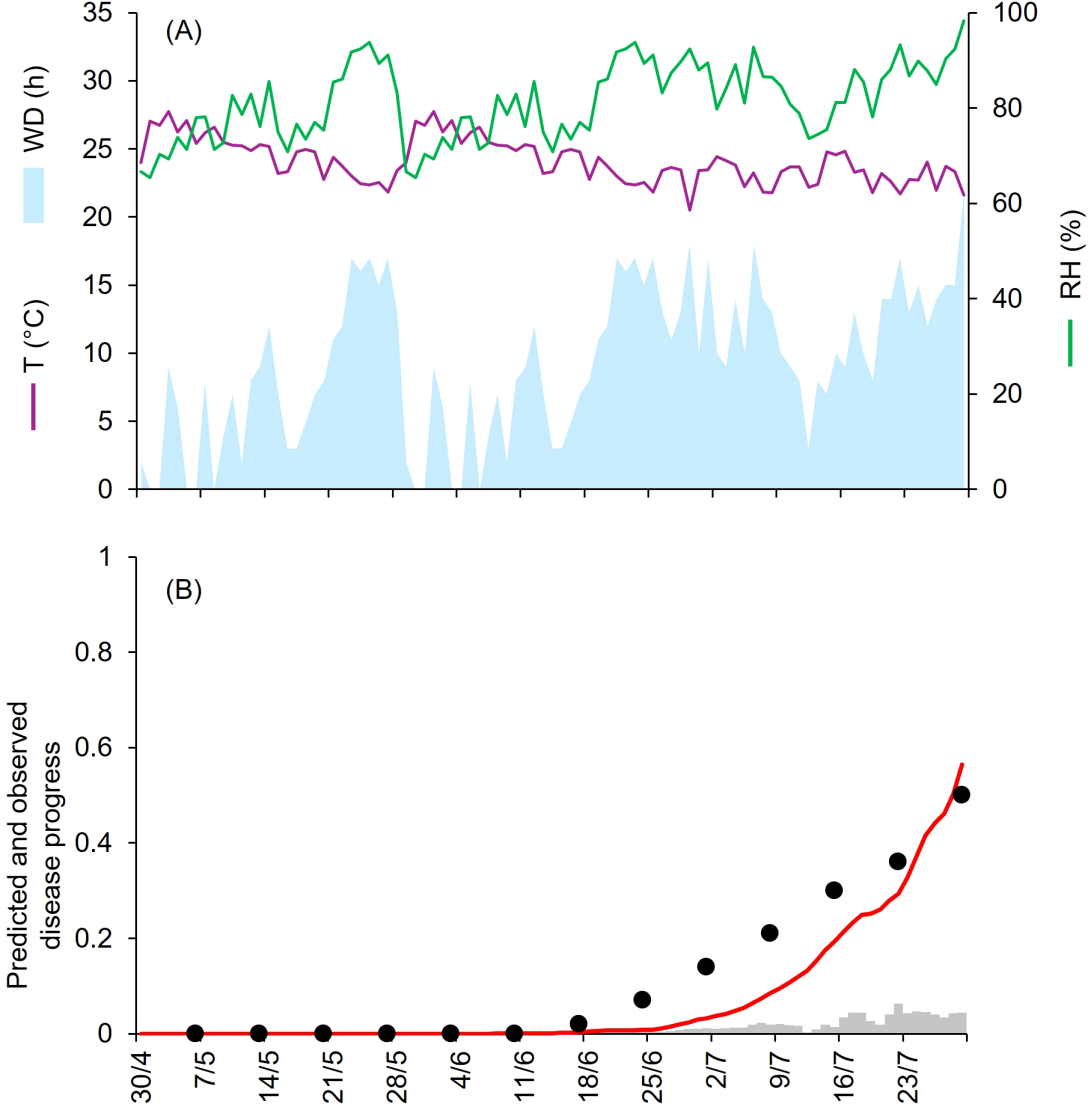
Predicted and observed disease progress at Bengalore, KA, India, in 2016 (KA-16). **(A)** Weather variables: air temperature (T, in °C; purple line), relative humidity (RH, in %; green line), and wetness duration (WD, in hours; light blue area). **(B)** Infection severity predicted by the model (light gray bars), disease severity predicted by the model (red line), and observed disease incidence (black dots).

At KA-12 to KA-15, epidemics developed from the end of June to mid-October, with final disease severity from 86% to 100% (**Table 1**). At KA-12 and KA-13, the growing season was mild and humid, with daily av. T, RH, and cumulated WD hours of 23.9°C, 72.3% and 374 h, and 23.4°C, 77.8%, and 478 h, respectively (**Figures 5A**, **6A**). Instead, at KA-14 and KA-15, the growing season was warm and wet, with daily av. T, RH, and cumulated WD hours of 27.9°C, 75.3% and 652 h, and 28.0°C, 81.2%, and 1369 h, respectively (**Figures 7A**, **8A**). For all these epidemics, both observed and predicted disease severity increased gradually over time (**Figures 5B** to **8B**), resulting in CCC ≥ 0.982 and a RMSE ≤ 0.075. Negative CRM values (–0.054 and –0.029) indicated slight tendency toward overestimation. At KA-16, disease assessment was performed between May and July, when disease severity reached 50%. A mild and wet weather (av. T = 24.1°C, av. RH = 81.2%, and cumulated WD = 839 h; **Figure 9A**) triggered the beginning of the epidemic in mid-June; afterwards, the disease increased gradually (**Figure 9B**) reaching higher levels of severity than those recorded in July for the four previous years. For the goodness-of-fit of predicted versus observed data, CCC = 0.927 and RMSE = 0.063. The model showed a tendency toward underestimation (CRM = 0.262), likely due to a delay in the prediction of first seasonal infection that lagged the predicted disease progression of about one week.

**Figure 10:**
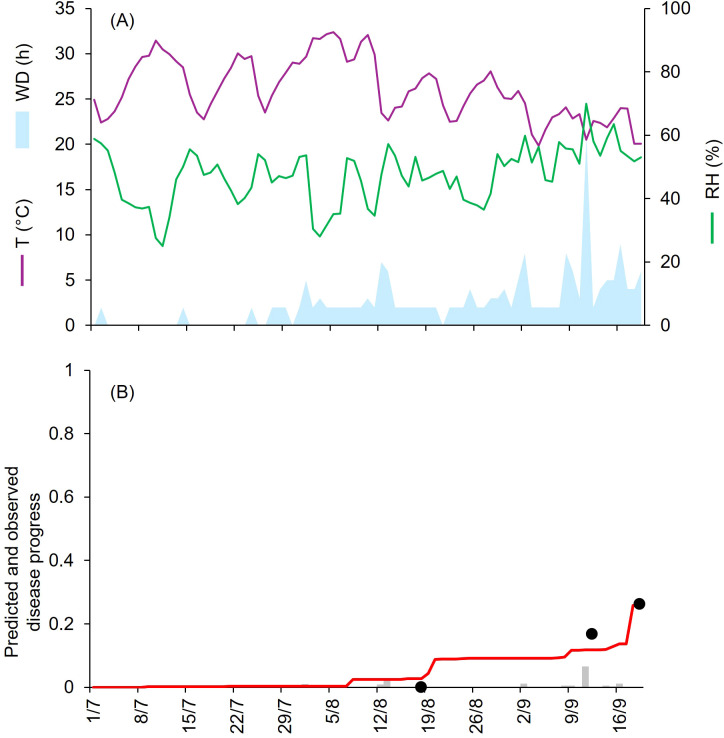
Predicted and observed disease progress at Foggia, Italy, in 2017 (IT-17). **(A)** Weather variables: air temperature (T, in °C; purple line), relative humidity (RH, in %; green line), and wetness duration (WD, in hours; light blue area). **(B)** Infection severity predicted by the model (light gray bars), disease severity predicted by the model (red line), and observed disease incidence (black dots).

**Figure 11:**
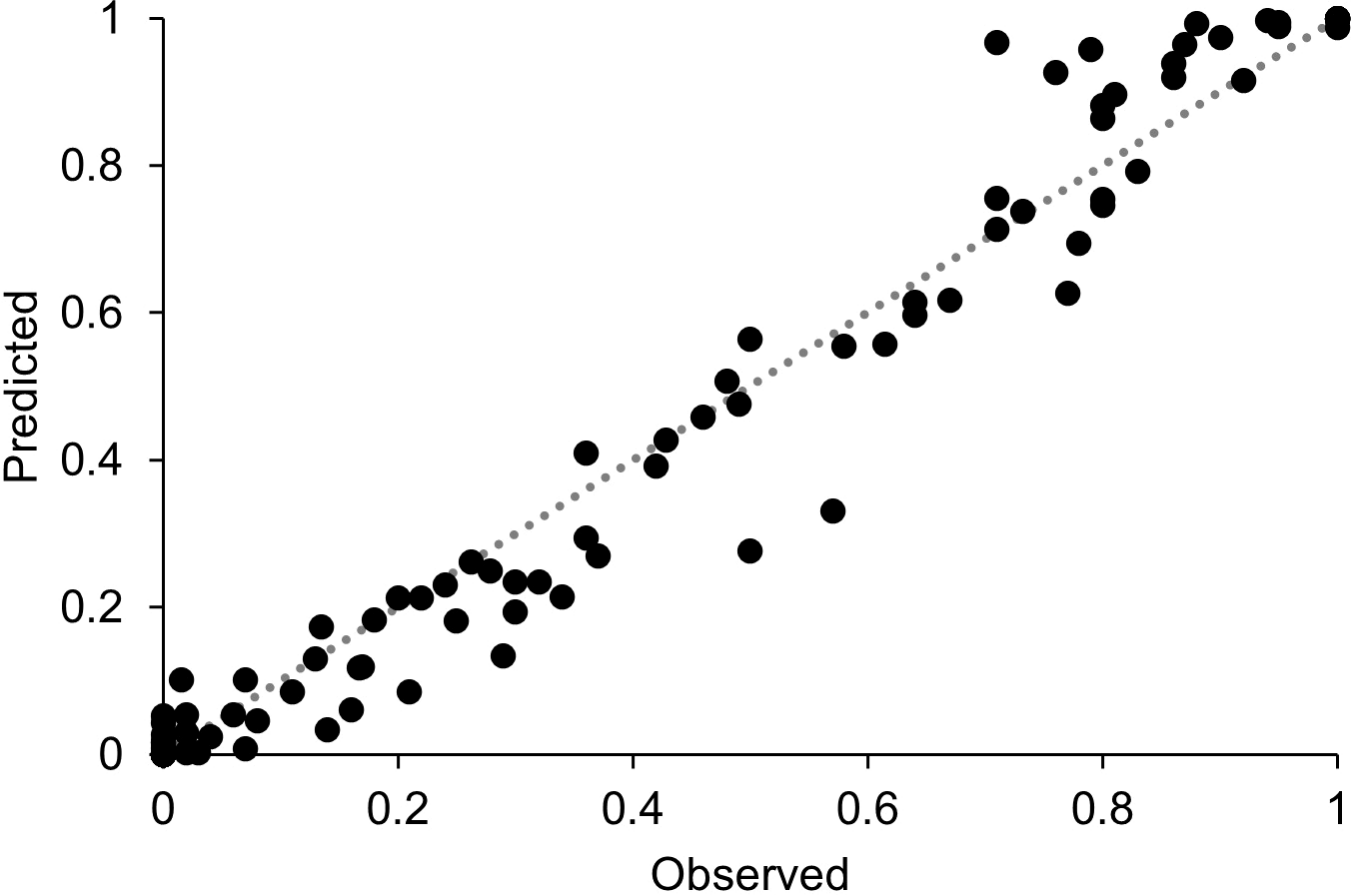
Predicted versus observed values of disease severity in the locations and years listed in **Table 1**; the dotted line is the perfect agreement line.

At IT-17, the model ability in negative prognoses can be observed. In fact, a warm and dry season (av. T = 26.1°C, av. RH = 47.1%, and cumulated WD = 194 h; **Figure 10A**) led to the outbreak of the disease only in mid-September, with final severity of 26% (**Table 1**). The model predicts few infections in mid-August, which resulted in visible symptoms at the end of the month, and repeated infection from the beginning of September leading to an exponential progress of the disease (**Figure 10B**). A CCC = 0.95 and a RMSE = 0.032 indicated good agreement between observed and predicted data; a CMR = 0.055 indicated a slight tendency toward underestimation. An overall comparison of predicted versus observed data (**Figure 11**) indicated a good agreement between the fitted line and the perfect agreement line (CCC = 0.982), with little average distance between real data and the fitted line (RMSE = 0.069). The model showed a slight tendency toward underestimation (CRM = 0.015).

## 4 Discussion

This study presents the development of ALT-tomato, which is a mechanistic, weather-driven model describing *Alternaria* epidemics in tomato and explicitly linking infection dynamics with potential mycotoxin risk. The framework integrates key epidemiological processes, primary inoculum activation, secondary infection cycles, and environmental modulation of pathogen activity, within a multi-species context. The model output was evaluated against independent epidemic datasets characterized by contrasting environmental conditions and it consistently reproduced the main temporal patterns of disease development, with agreement metrics indicating a satisfactory balance between accuracy and bias across scenarios. Notably, model performance remained stable across epidemics differing in onset timing and overall severity, supporting the robustness of the selected process-based structure.

The ability of the model to reproduce epidemic dynamics across different environmental conditions suggests that *Alternaria* epidemics at the field scale are largely structured by environmental forcing. Under the conditions examined, disease development emerges from the interaction between biological processes and environmental forcing, with environmental conditions shaping the expression and timing of key biological mechanisms explicitly represented in the model. This view is consistent with previous experimental and modeling studies that highlight the dominant role of environmental forcing in polycyclic fungal epidemics (Pautasso et al., 2012; Savary et al., 2019) and provides a coherent framework for interpreting the assumptions adopted in the model. The Alternaria disease complex on tomato was represented through *A. alternata*, *A. solani*, and *A. tenuissima*, reflecting their frequent association with tomato epidemics and post-harvest infections and their importance in epidemiological and toxicological studies (Thomma, 2003; Woudenberg et al., 2015; Pinto and Patriarca, 2017). Although *A. alternata* and *A. tenuissima* are phylogenetically close and share similar temperature range for biological processes, they diverge in the temperature-dependent response (as revealed by a different parametrization of model equations), especially for mycotoxin production. In fact, *A. tenuissima* shows a lower optimal temperature (around 20°C) compared to *A. alternata* (around 30°C), as described by the temperature-dependent function in Equation 13 derived from Salotti et al. (2025b). Maintaining this species resolution in the model framework might better support the prediction of mycotoxin risk; nevertheless, further studies and model validation for mycotoxin contamination are required to support this hypothesis.

Although additional *Alternaria* species have occasionally been isolated from tomato tissues, their epidemiological relevance remains poorly quantified and species-specific data suitable for mechanistic parameterization are largely unavailable, making it difficult to assess whether such taxonomic detail would substantially improve epidemic-scale predictions. In this context, the satisfactory model performance across independent epidemic datasets suggests that, at the spatial and temporal scales considered, epidemic development is primarily governed by environmentally driven processes shared across *Alternaria* species, a conclusion also supported by the fragmented and species-imbalanced nature of available biological data reported in recent systematic review (Salotti et al., 2024).

This interpretation is reinforced by the limited loss of performance when species-specific details were aggregated, with high concordance correlation coefficients and low RMSE values across independent epidemics. This indicates low inter-species variability in the response to environmental drivers at field scale. This interpretation is consistent with evidence of limited inter-specific variability in temperature- and relative humidity-dependent responses for key processes such as growth, infection, and secondary metabolism (Rotem, 1994; Andersen and Frisvad, 2002). In addition, it is aligned with findings from other pathosystems where simplified representations of pathogen population structure did not compromise model robustness (Magarey et al., 2005; Rossi et al., 2008). Taken together, these considerations support a modeling strategy in which biological details are selectively simplified when its contribution to epidemic-scale dynamics is expected to be limited. Within this frame, the exclusion of chlamydospore formation, reported mainly for *A. solani*, represents a sustainable deliberate modeling choice. Although chlamydospores may contribute to long-term survival, their role in driving within-season epidemic dynamics remains unclear, and comparable survival structures have not been consistently documented for other *Alternaria* species affecting tomato Agrios (2005); Thomma (2003); Rotem (1994). Similarly, the assumption of an equal contribution of the three modeled species reflects the absence of reliable data on species composition and temporal dynamics in field populations. The limited impact of this assumption on model performance suggests that aggregate infection pressure, rather than species-specific prevalence, is the primary determinant of disease dynamics under the conditions examined. Furthermore, all the considered species are able to produce AOH, AME, and TeA, therefore, their relative proportions may not provide substantial additional information. In this sense, and given that toxin accumulation is directly linked to the intensity and timing of infection processes, this assumption provides a coherent basis for extending epidemic modeling toward food safety-oriented outputs.

The integration of a toxin-oriented module extends Alternaria disease complex modeling beyond infection predictions and reflects increasing interest in linking plant disease epidemiology with food safety outcomes. Previous studies have shown that the occurrence of toxins produced by *Alternaria* spp. in tomato is strongly influenced by infection timing, environmental conditions, and host susceptibility, rather than by symptom occurrence alone (Logrieco et al., 2009; Patriarca, 2016; Pinto and Patriarca, 2017). Accordingly, the model supports the interpretation of toxin risk as an outcome of epidemic development. The consistency of predicted infection dynamics provides a necessary precondition for interpreting toxin-related outputs, as the reliability of the toxin module directly depends on the robustness of the underlying epidemiological predictions.

Building on this epidemiological framework, the focus on AOH, AME, and TeA reflects their frequent detection in tomato and their central role in current human exposure and risk assessments from a regulatory perspective (EFSA Panel on Contaminants in the Food Chain (CONTAM), 2011, 2016). This relevance is further supported by EU monitoring guidance, as reflected in Commission Recommendation (EU) 2022/553, which proposes occurrence thresholds for *Alternaria* toxins in selected food commodities, including processed tomato products, to support harmonized surveillance across Member States. By contrast, although *Alternaria* species can produce a wide range of secondary metabolites, TeA, AOH, and AME are among the toxins most consistently reported along the tomato chain, commonly detected in fresh tomatoes and frequently found in processed tomato products (Sanzani et al., 2019; Qin et al., 2022; Ackermann et al., 2011; Bertuzzi et al., 2021). For several other *Alternaria* metabolites, evidence in tomato matrices is comparatively limited and/or less consistent, and where reported, often lacks the temporal and environmental resolution (Logrieco et al., 2009; Ostry, 2008). Conversely, the production of AOH, AME and TeA has been repeatedly shown to respond in a systematic manner to environmental factors, particularly temperature, providing a clearer basis for linking epidemic development with toxin dynamics (Andersen et al., 2015; Salotti et al., 2024).

Although the epidemiological component of the model was evaluated against independent field datasets and showed robust agreement with observed disease dynamics, direct validation of toxin-related outputs was not possible due to the scarcity of field datasets that simultaneously reported disease severity, species composition, geographical coordinates, weather data, and quantitative toxin measurements. In this context, the consistency between predicted infection dynamics and the epidemiological drivers known to influence *Alternaria* toxin occurrence supports the interpretation that the model captures first-order determinants of toxin risk through its validated representation of epidemic dynamics. The toxin module should therefore be regarded as a hypothesis-driven component, intended to support risk prioritization and guide targeted monitoring rather than to provide quantitative predictions. In this sense, the proposed framework provides a structured basis on which emerging field datasets that integrate disease progression and mycotoxin measurements can be directly exploited for further refinement and validation of the model (Salotti et al., 2024).

Overall, the results indicate that simplified, process-based representations can effectively describe *Alternaria* epidemics at the field scale, provided that dominant environmental drivers are adequately captured. Rather than aiming at species-resolved prediction, ALT-tomato offers a biologically informed structure to integrate epidemic dynamics with food safety considerations under variable environmental conditions.

The explicit link between disease development and potential toxin risk highlights the value of epidemiologically grounded approaches to support risk prioritization and surveillance strategies. At the same time, the analysis clearly identifies critical data gaps, particularly the lack of integrated field datasets that combine disease progression, species composition, and quantitative toxin measurements. Addressing these gaps will be essential to further validate and refine toxin-oriented modeling components and advance decision-support tools at the interface between plant disease management and food safety.

## Data Availability

The original contributions presented in the study are included in the article/supplementary material. Further inquiries can be directed to the corresponding author.
